# MyCites: a proposal to mark and report inaccurate citations in scholarly publications

**DOI:** 10.1186/s41073-020-00099-8

**Published:** 2020-09-17

**Authors:** Mohammad Hosseini, Martin Paul Eve, Bert Gordijn, Cameron Neylon

**Affiliations:** 1 Institute of Ethics, School of Theology, Philosophy, and Music, Dublin City University, Dublin, Ireland; 2 Department of English and Humanities, Birkbeck University of London, London, UK; 3 Centre for Culture and Technology, Curtin University, Perth, Australia

**Keywords:** Inaccurate citations, Editorial process, Post-publication peer-review, Annotations, Research integrity, Responsibilities

## Abstract

**Background:**

Inaccurate citations are erroneous quotations or instances of paraphrasing of previously published material that mislead readers about the claims of the cited source. They are often unaddressed due to underreporting, the inability of peer reviewers and editors to detect them, and editors’ reluctance to publish corrections about them. In this paper, we propose a new tool that could be used to tackle their circulation.

**Methods:**

We provide a review of available data about inaccurate citations and analytically explore current ways of reporting and dealing with these inaccuracies. Consequently, we make a distinction between publication (i.e., first occurrence) and circulation (i.e., reuse) of inaccurate citations. Sloppy reading of published items, literature ambiguity and insufficient quality control in the editorial process are identified as factors that contribute to the publication of inaccurate citations. However, reiteration or copy-pasting without checking the validity of citations, paralleled with lack of resources/motivation to report/correct inaccurate citations contribute to their circulation.

**Results and discussion:**

We propose the development of an online annotation tool called “MyCites” as means with which to mark and map inaccurate citations. This tool allows ORCID users to annotate citations and alert authors (of the cited and citing articles) and also editors of journals where inaccurate citations are published. Each marked citation would travel with the digital version of the document (persistent identifiers) and be visible on websites that host peer-reviewed articles (journals’ websites, Pubmed, etc.). In the future development of MyCites, challenges such as the conditions of correct/incorrect-ness and parties that should adjudicate that, and, the issue of dealing with incorrect reports need to be addressed.

## Introduction

Within all areas of research, the use of previously published material is an essential building block of knowledge-production [1]. This is so indispensable that, famously, Isaac Newton described the process of discovering the truth by relying on previous explorations as standing on the shoulders of giants [2]. However, research results are sometimes used sloppily and cited inaccurately.

Inaccurate representations of previously published material can mislead readers about the claims of the cited source, and distract the accurate flow of information and history of ideas. This pertains not just to bibliographic errors such as spelling mistakes in authors’ names or an incorrect publication year, but to erroneous quotations and misleading paraphrases. While both bibliographic errors and misleading quotations/paraphrases are problematic, this paper only focuses on the latter issue and refers to this phenomenon with the term *inaccurate citation*.

## Inaccurate citations, their scale and the parties involved

Several studies have gauged inaccuracies in citations within a particular field. For instance, Todd et al. analysed 306 citations in the field of Ecology and concluded that in 11.1% of the citations, “the cited article has been interpreted one way, but could also be interpreted in other ways – including the opposite point” [3]. In 7.2% of the citations, however, “the cited article did not in any way substantiate the assertion or results attributed to it” [3]. In their meta-analysis, Jergas and Baethge review the results of 28 studies - published between 1985 and 2013 - on inaccurate citations in peer-reviewed medical journal articles. Reporting on the analysis of 7321 citations, the worrisome conclusion is that 11.9% of citations have major errors (not at all in accordance with what the cited authors claimed), and an additional 11.5% have minor errors (“inconsistencies and factual errors not severe enough” to contradict a statement by the cited authors) [4]. Most of the 28 studies considered for meta-analysis chose a sample that involved citations of multiple sources. Studies that focus on citations of a single target source yield much more troublesome results.

Authors who manually checked how their publications are being cited and used in other studies report much higher rates of inaccuracies and provide classical examples of inaccurate citations for our analysis. In 2018, for instance, Stang et al. analysed a random sample of 100 publications that cited a 2010 paper that Stang had published about the Newcastle-Ottawa scale (a scale used to judge the quality of observational studies in systematic reviews). While Stang (2010) criticises this scale [5], 94 out of 100 randomly selected articles that cited Stang’s study claimed that the 2010 paper *supported* the use of this scale [6]. In a similar exercise, Glenton and Carlsen assessed 205 articles that cited their 2011 paper about sample sizes in focus groups [7]. They found that in 50.7% of citing articles, their descriptive report about typical sample sizes was being used as a *normative* justification for the sample size in studies that cited it [8]. According to the definition used in Jergas & Baethge’s meta-analysis, both mentioned examples demonstrate a worrisome percentage of *major* quotation errors. Authors of the above-mentioned studies suspect that their articles were poorly read or not read at all, which echoes the claim that some researchers do not read what they cite and merely copy and paste claims and citations from other papers [9–11].

Considering other contributing factors, cited authors may also be partially responsible for inaccurate citation of their works through their biased reporting of results in abstracts [12, 13] or biased use of language that may have confused readers [14, 15]. Furthermore, neither peer-reviewers nor editors are always able to prevent the publication of articles with inaccurate citations; an aspect mentioned by authors of both studies who checked how they had been cited [6, 8]. It may even be unreasonable to expect peer-reviewers who mostly provide their service for free, and editors who often have to manage a massive manuscript flow and are rarely well-compensated, to check every single citation for its accuracy. Given the challenges of spotting all inaccuracies during the editorial process in advance of publications, we believe it is useful to make a conceptual distinction between the *publication* and the *circulation* of articles containing inaccurate citations. This distinction allows us to analyse the role of more parties and consider new solutions.

The publication of an inaccurate citation refers to the first time that a resource is referenced inaccurately (Case zero). The circulation of an inaccurate citation, however, pertains to the propagation of the already existing inaccurate citations. This is done by researchers who take the inaccurate interpretation/assertation of *Case zero* for granted, and without a proper reading of the original item, reiterate, rephrase, or simply copy-paste inaccurate citations.

**Fig. 1:**
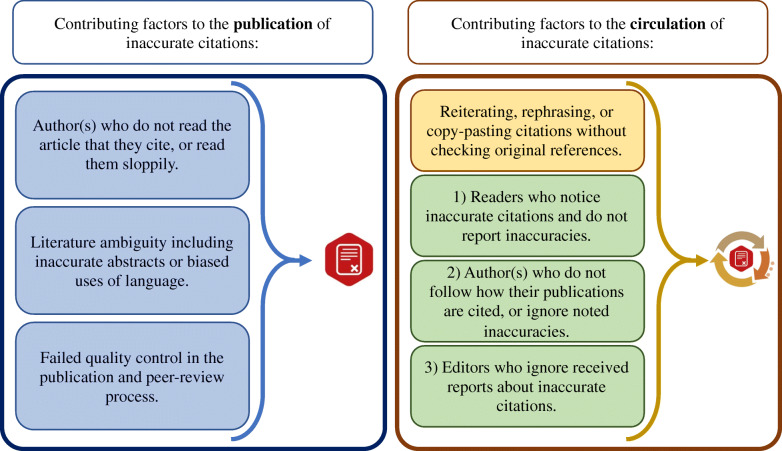
Contributing factors to the publication and circulation of articles with inaccurate citations. Green boxes represent some parties that could prevent the propagation of inaccurate citations but often do not do this

Whilst it is difficult to completely prevent the publication of papers with inaccurate citations, it is worthwhile to focus on tackling their continued circulation. Shifting the focus from the publication to the circulation allows us to consider the role played by a range of parties that could prevent the propagation of inaccurate citations, but do not do this. While it is true that researchers who copy-past inaccurate citations without reading the original reference make a major contribution to the circulation of inaccurate citations, other parties may contribute to this problem with their inaction (see green boxes in Fig. 1). Some of these parties include (1) readers who notice inaccurate citations, (2) authors who are being cited inaccurately and (3) journal editors.

1. Informed readers are increasingly expected to be proactive about reporting errors in the literature [16]. However, since commensurate reward structures to incentivise raising concerns about inaccurate citations have not emerged, this is not common practice.
2. It is reasonable to argue that among the post-publication responsibilities of authors of the cited article, one is to react to inaccurate citations of their work. If authors such as Stang, and, Glenton and Carlsen had not reacted to inaccurate citations of their work, they would have contributed to further circulation of inaccurate citations.
3. Among the post-publication tasks of editors, one pertains to their response to reported concerns about the soundness of published items [17]. Nevertheless, engaging with reports about inaccurate citations may not always be their top priority. In a famous example, editors of the journal of *Experimental Economics* noted in an editorial:
  “As a general rule, the journal in the future will not publish errata to an article merely to point out omitted, inaccurate, or inappropriate, citations. Exceptions may be made to this rule, but we intend these to be very rare. Those authors who feel that they should have been cited or that were cited inaccurately in an article that we have published will have to use other means, such as posting notices on their own websites or contacting key researchers doing related work directly, to notify the research community of their concerns” [18].

While it is likely that other factors contribute to publication and circulation of inaccurate citations (e.g., production errors, honest errors, intentional misue of original information or the creation of fake news), to the best of our knowledge, there have not been any large studies exploring them and their importance or relevance in different disciplines. Regarding the particular case of editors, in the absence of qualitative research that explores their willingness to engage with this issue, or quantitative studies that compare the number of reported inaccurate citations against published errata, further analysis of editors’ behaviour cannot be provided. Nevertheless, since inaccurate citations are not among classical instances of misconduct, and, correcting them involves protracted communication between the editor and several parties; one understands why editors might not be keen on engaging with inaccurate citations.

## Current ways of reporting inaccurate citations

A summary of current ways of reporting inaccurate citations is presented in Table 1, and described in detail below. To correct inaccurate citations, they must first be located and reported. However, the process of reporting inaccurate citations has major problems that might actually demotivate those who spot them from taking any action and thus allowing them to remain in circulation. Without attempting to be exhaustive, in what follows six options for reporting inaccurate citations, and problems associated with each are introduced.

1. Contacting the editor of the journal where the publication with an inaccurate citation was published involves finding editors’ contact information and sending an email to them (with the details of the article, page number, paragraph, citation, the cited article, and a description of inaccuracies), all of which is time-consuming. Given the prevalence of inaccurate citations, contacting the editors for each incidence is an inefficient way of reporting errors.
2. One can submit a commentary/letter about the error to the journal where the paper with an inaccurate citation is published. Readers have been encouraged by their peers for many years to make use of the *Correspondence* or *Comments* sections of journals to “point out and correct misleading quotation errors” [19]. Preparing and publishing a letter/comment is time-consuming, both for authors and the editorial team. If the inaccurate citation has not been the linchpin of a paper, this could be considered as a well-intentioned but inordinate act that overburdens the editorial team. It would be extremely inefficient to publish a letter/commentary for every single inaccuracy.
3. Reporting inaccuracies with an email to the corresponding author of the cited and/or citing article, and allowing them to decide how to proceed is another possibility. Even if this report is not ignored, it is very likely for the reporter to be left outside of the communication loop. In which case, the reporter cannot know what happens with their report and whether it is being acted upon or not. If authors from citing and cited papers get in contact with each other, they may just agree that inaccurate citations should not be repeated in the future and they may never contact the journal or submit a corrigendum.
4. One can report inaccuracies via social media platforms or other outlets that promote(d) the publication with inaccurate citation. Especially with the help of applications such as Altmetrics, it is possible to find mentions of publications on Twitter, blogs and news outlets. One can post (publicly visible) comments to report inaccuracies. In addition to the possibility of having fake/anonymous/pseudonymous names (that may complicate distinguishing a genuine report from trolls), these platforms often allow account owners to delete unwanted comments. Furthermore, not every researcher is a member of these platforms and given the presence of a wide range of non-experts, laypersons, family and friends, social media is perhaps the worst possible place to report inaccurate citations.
5. One can make a comment on post-publication platforms such as PubPeer. Although useful for post-publication conversations, PubPeer is not designed for the purpose of reporting inaccurate citations. Prose comments about an article in its entirety, are not linked to citations, which has two drawbacks. Firstly, cited authors will not be notified about the inaccuracy, and secondly, it would not be possible to interrogate the use of a source and notify future readers about an inaccurate citation where it occurs in the body of the paper. Furthermore, PubPeer seems to be moving away from an open and free access model towards a hybrid subscription model, which ristricts users’ access to functionality regarding comments. Their new dashboard that allows creating efficient feedback loops and searching through comments is designed to serve journals and institutions for a price [20]. Another complexity of using PubPeer for this purpose is the possibility of making anonymous comments. While this functionality might be useful at times, it has also contributed to disputes and controversies [21], and may further complicate managing reports and distinguishing genuine ones from trolls.
6. Concerns can also be voiced via limited annotation capabilities offered by preprint servers and some journals. The journal of *eLIFE*, for example, have joined forces with Hypothes.is annotation software and offer annotating capabilities on their website [22]. While innovative and useful, these comments will not be visible to readers who may prefer to read an article on other platforms such as PubMed. Furthermore, these prose annotations are not searchable or linked to citations with digital identifiers. This drawback would not allow having the cited authors notified about the inaccuracy, or generate an index of inaccurate citations.

**Table 1:** Possible outcomes and problems of reporting inaccurate citations using current methods

Method	Possible outcomes	Problems
1. Contacting the editor	• Publication of a corrigendum • No correction, editor contacting authors with a note to be more careful in the future • Being ignored	• Burdensome for the reporter and the editoral team • Impractical to do for every single inaccuracy
2. Submission of a commentary/letter	• Acceptance of commentary/letter and publication of a corrigendum • Acceptance of commentary/letter and publication of a response commentary/letter by the citing authors in reaction to the first letter with/without the publication of a corrigendum • Acceptance of commentary/letter without any further response from the citing authors, or reaction from the editors • Rejection of the commentary/letter	• Burdensome for the reporter and the editorial team • Impractical to do for every single inaccuracy
3. Email corresponding authors	• Submission of a corrigendum by authors and having the inaccuracy corrected • Submission of a corrigendum and having it rejected by the editors • Being ignored	• The reporter may remain out of the communication loop • The inaccuracy may never be reported to the journal
4. Post on social media	• Submission of a corrigendum by authors and having it corrected • Submission of a corrigendum and having it rejected by the editors • Being ignored • Having the comment deleted	• Complicated to distinguish genuine report from trolls • Comments can be deleted by account owners • Scientists who are not on social media will not be aware of it • Exposure to non-experts
5. Use PubPeer	• Editors of the journals with a *paid* subscription will get a notification. They may react with/without a corrigendum, or ignore the report • Citing authors act on the reported inaccuracy and submit a corrigendum which may be accepted or rejected • Citing authors ignore the report • Other researchers including the citing/cited authors may make further comments or ignore it altogether	• Prose comments do not appear in the text • Comments are not linked to citations • Cited authors are not notified • Not fully open and free for all users • Possible to make anonymous comments
6. Current annotation platforms	• Editors of the journals who wish to be informed will get a notification. They may react with/without a corrigendum, or ignore the report • Citing authors act on the reported inaccuracy and submit a corrigendum which may be accepted or rejected • Citing authors ignore the report • Other researchers including the citing/cited authors may make further comments or ignore it altogether	• Comments are not visible across platforms • Comments are not linked to citations • Cited authors are not notified • Not possible to create an index of inaccurate citations

## Towards a new solution

**Fig. 2:**
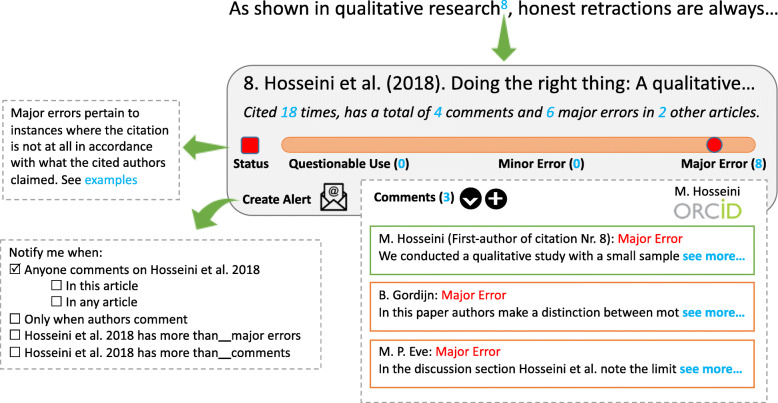
A graphical prototype of MyCites tool that would appear by clicking on an in-text citation

**Fig. 3:**
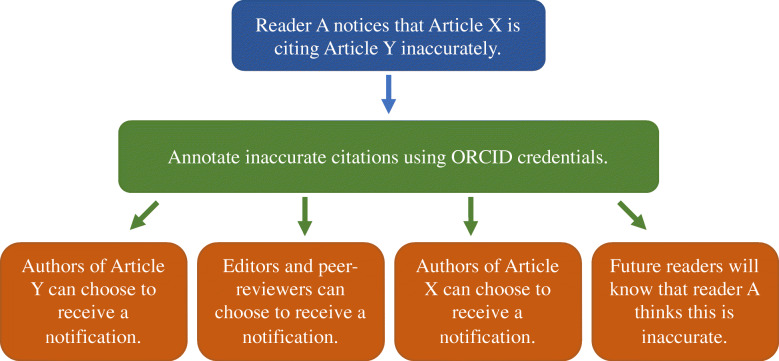
Annotating inaccurate citations benefits various parties and streamlines the process of reporting errors

Against the backdrop of the current problems of reporting inaccurate citations, it is pivotal to learn from the shortcomings of current practices in order to simplify and streamline this process. We propose and are piloting a new tool that consolidates annotation capabilities (such as those of Hypothes.is) with persistent identifiers (such as ORCID), as well as Open Citation Identifiers (OCI) [23] and In-Text Reference Pointer Identifier (InTRePID) [24], to simplify locating and marking in-text citations. We call this prototype MyCites (see Fig. 2) and welcome correspondence from those who wish to be involved in this endeavour (For further technical explanation about OCI and InTREPiD, and how they can be used to generate a new persistent identifier, see the supplementary file. MyCites is a provisional title for the prototype and the project. We might choose a different name once the tool is developed). We believe that such a tool needs to allow readers, editors and also authors who are cited inaccurately, to receive notifications (see Fig. 3).

Designing a devolved mechanism to annotate inaccurate citations has four advantages. First, by creating a space to raise concerns about inaccurate use of citations, it might deter malpractice. Second, it streamlines the process of highlighting and reporting inaccurate citations. Third, it notifies new readers about inaccurate citations that are spotted by previous readers. Finally, in case it is effectively adopted and used by researchers, it could prevent the continued circulation of inaccurate citations and improve the accuracy of links between citing and cited items.

Allowing ORCID users to (publicly) post personal views about citations is not without challenges, and some of these are mentioned below.

### The definition of inaccurcy

It is crucial to develop a theoretical grounding and shed more light on the theoretical/conceptual question of what it means for a citation to be ‘(in)accurate’. It is also necessary to create a taxonomy of the different kinds of inaccurate citations.

### Adjudication and arbitration

Given the importance of defining what a correct citation is, identifying one person to have the authority to adjudicate correctness and arbitrate possible disputes could become very complicated. For instance, in cases where the cited paper is co-authored by researchers who may disagree with each other about the best interpretation of their work, someone else would have to decide what interpretation of their work is accurate. Furthermore, in cases where the author(s) are no longer alive, choosing one person to indicate what they meant would be very challenging (especially in Humanities and Arts where one can make alternative interpretations of the text).

### Responsibility and accountability

Considerations about responsibility and accountability with regards to inaccurate citations are particularly challenging to address. For example, when inaccuracies result in significant losses (e.g., resources, lives), or, in cases where the cited article makes a biased use of language, or, is poorly written and convoluted, pointing the finger at one person would be challenging. Especially in cases where more than one co-author has been involved in the development of the text, responsibilities are diffused.

### Reliability of annotations

It is likely that not every reported inaccuracy is reliable and valid. Addressing this issue in different ways will affect the number of reports and the availability of the tool. For example, one way of ensuring that reported inaccuracies are reliable is to have them confirmed by others (e.g., similar to the peer-review processes where two/three referees are involved). Another approach could be to limit the access of researchers from outside a particular discipline to mitigate the risk of misuse, trolling and wasting people’s time through irrelevant complaints.

### Incorrect reports and permanence of comments

There may be compelling reasons for removing/hiding incorrect annotations. In this case, someone (e.g., the cited author or the editor) would have to make this decision. Besides the complexity of indicating one person with authority to remove/hide annotations, this also raises questions about the specific conditions that should be met for an annotation to become removed/hidden.

### Legal implications and requirements

Although considerations about the need for curation or moderation of annotations seem more relevant to upkeep and maintenance, in cases that annotations are used to blackmail or defame the competition, legal aspects stand out. Should annotations be immediately live and visible to everyone, or is the use of a *vulnerability disclosure model* (public disclosure of issues only after a certain period) more acceptable? For example, annotations could be first made visible to main parties such as the citing and cited author as well as journal editors to give parties with major stakes some time to react. How and where will the data be stored, who should own this data and for how long, are among questions that would impact copyrights and the use of comments in the future [25].

### Application to preprint servers and journals that offer annotation capabilities

Preprints are increasingly part of the recognised scientific output. Integrating a new tool into preprint serves, and journals that use other annotation technologies might be challenging. Furthermore, it is not clear whether preprint servers and other journals that allow articles to have a version-number should issue a new version after correcting a citation.

### Citation indices, citation identifiers and pointers

This tool needs to be linked with a citation index, and accordingly, will have limitations based on the comprehensiveness and openness of the chosen index (although projects such as Open Citations might gradually resolve this in the future [26]). Similarly, OCIs and InTRePIDs do not capture all citations yet, which might add further limitations to the coverage. Furthermore, interpreting the link between OCIs, InTRePIDs, and annotations about inaccurate citations might not always be straightforward. For example, if one paper is cited three times (for three different reasons) in another paper, and only one of those citations is inaccurate, a simple index might not be able to capture such complexity.

### The indispensability of a new tool

One might question why a new tool is needed at all in the presence of post-publication peer review applications such as PubPeer, and annotation software applications such as Hypothes.is. We believe that although both PubPeer and Hypothes.is have been successful in achieving their goals, neither is designed to tackle the problem of inaccurate citations. Other initiatives focused solely on citations (e.g., Scite.AI that use artificial intelligence to clarify whether a citation provides supporting or contradicting evidence for the cited claim [27]), might be very effective in detecting bibliographic errors. However, these tools too are not designed to detect incorrect citations that pertain to erroneous quotations or paraphrases and given semantic complexities of identifying these errors; it is not clear whether artificial intelligence should be used for this purpose at all.

### Engagement, uptake and impact

Finally, one might ask whether researchers would care enough to engage with such a system and if yes, what would be the real impact? The engagement of scientists with new tools and resources that are meant to improve the integrity of published research and the impact of these initiatives have indeed shown inconsistencies. For instance, even after the launch of Retraction Watch database in 2018 [28], and the integration of retracted articles into reference management systems such as Zotero [29], citation of retracted articles has not stopped [30]. Furthermore, while PubPeer is used by experts who are heavily involved in exposing errors, for example, Dr. Elisabeth Bik (Image forensic expert who searches the biomedical literature for inappropriately duplicated or manipulated photographic images), not every author or editor responds to spotted and reported errors [31]. Without misleading ourselves into thinking that a new tool that allows annotating inaccurate citations would not face similar problems, we believe that more research is needed on this topic. In the absence of a final prototype and empirical data about researchers’ expectation and feedback, it is difficult to theorise about the question of engagement and uptake by the research community.

## Conclusion

Inaccurate citations are prevalent to a worrisome degree. While the true impact of inaccurate citations and their effect on knowledge production and research waste remain difficult to assess, current methods of reporting them are largely inefficient and problematic. Hence, they keep circulating in the literature. We believe that this vicious cycle could be slowed down, and suggest a solution that, if adopted and used by researchers, could be helpful in containing the circulation of inaccurate citations.

By utilising available capacities of the modern publishing landscape, we are developing MyCites to annotate inaccurate citations. We believe that since the ultimate owners of the problem of inaccurate citations are members of the scientific community, they should be involved from early stages of developing MyCites. Currently, we are setting up a taskforce with two working groups to further develop this tool. One working group focuses specifically on the conceptual issues, and the other on technical development. Hence, we welcome correspondence from those who can help us with conceptual/technical aspects of this endeavour. The two working groups will work in tandem and be coordinated by the chairing board (authors of this article). We also welcome suggestions for partnership and co-development of this tool.

## Supplementary information

**Additional file 1.** Technical explanation about OCI and InTREPiD, and how they can be used to create a new persistent identifier.

## Data Availability

Not applicable.
