# Supplementary material for: MyCites: a proposal to mark and report inaccurate citations in scholarly publications
Source: Res Integr Peer Rev. 2020 Sep 17;5:13. doi: 10.1186/s41073-020-00099-8 (PMC7500547; doi:10.1186/s41073-020-00099-8)
Supplement: Supplementary file 1 — Additional file 1. Technical explanation about OCI and InTREPiD, and how they can be used to create a new persistent identifier. [file 41073_2020_99_MOESM1_ESM.docx]

Technical explanation about OCI and InTREPiD, and how they can be used to create a new persistent identifier

Both OCI and InTRePID are developed by the Open Citations project, formally based at the University of Bologna. OCIs ^[23]^ are unique persistent identifiers for citations themselves, treated as first-class data entities. They encode identifiers for the citing and cited publications prefixed with a supplier prefix (defined at https://github.com/opencitations/oci/blob/master/suppliers.csv) that specifies the source of the metadata about these publications. For example, oci:070433-070475 identifies a citation between two documents in the OpenCitations Citations in Context Corpus (supplier prefix 070) with identifiers “433” and “475”, respectively. For citing and cited documents identified by DOIs, each DOI is first transliterated from alphanumeric text into a pure numerical string, using the lookup table at https://github.com/opencitations/oci/blob/master/lookup.csv, before creating the OCI.

InTRePIDs^[24]^ permit the unique identifying of each in-text reference pointer (aka 'in-text citation') occurring at different points within the text that denotes a single bibliographic reference in the publication's reference list. The InTRePID is formed by taking the OCI for the citation to which that bibliographic reference is related, and adding a two-number suffix that identifies the relevant in-text reference pointer. Thus intrepid:070433-070475/4–6 represents the fourth in-text reference pointer out of a total of six that all denote the bibliographic reference related to the citation with OCI oci:070433-070475. The metadata associated with in-text reference pointers identified by InTRePIDs facilitate a more fine-grained analysis of citations, permitting, for example, analysis of the position in the document (e.g. Introduction, or Methods) and the textual context within which the in-text reference pointer is located, which may shed light on the author's purpose in making that citation at that particular point in the text.

By adding the annotator’s ORCID number as a prefix to the InTRePIDs, a new PID is created that allows the provenance of an annotation about a particular citation to be recorded (e.g., mycites:000000022385985X-070433-070475/4–6, where 000000022385985X is the annotator’s ORCID number).
